# Potential Indicators of Transition to Cognitive Decline in Older Men: A Longitudinal Analysis of Multiple Sensory, Balance, and Gait Changes

**DOI:** 10.3390/diagnostics16183026

**Published:** 2026-09-18

**Authors:** Jeongeun Yoon, Kiyoung Kwak, Dongwook Kim

**Affiliations:** 1 Department of Healthcare Engineering, Graduate School, Jeonbuk National University, 567 Baekje-daero, Deokjin-gu, Jeonju-si 54896, Jeonbuk-do, Republic of Korea; yje0546@jbnu.ac.kr; 2 Division of Biomedical Engineering, College of Engineering, Jeonbuk National University, 567 Baekje-daero, Deokjin-gu, Jeonju-si 54896, Jeonbuk-do, Republic of Korea; kykwak@jbnu.ac.kr; 3 Research Center for Healthcare & Welfare Instrument for Aged, Jeonbuk National University, 567 Baekje-daero, Deokjin-gu, Jeonju-si 54896, Jeonbuk-do, Republic of Korea

**Keywords:** cognitive decline, multiple sensory, dynamic balance, gait, MCI, dementia, generalized estimating equations

## Abstract

**Background/Objectives**: Early detection of cognitive decline is crucial for preventing dementia. Conventional diagnostic approaches, including neuropsychological test batteries, laboratory tests, neuroimaging, and cerebrospinal fluid analysis, may have limitations related to accessibility, cost, invasiveness, and the early detection of pathological changes. Dementia-related pathology can affect neural networks involved in sensory and motor functions, suggesting that changes in these functions may provide non-invasive indicators of subsequent cognitive decline. Therefore, this study aimed to investigate potential indicators associated with longitudinal cognitive decline by integrating multiple sensory and motor functions. **Methods**: Cognitive, sensory, balance, and gait function were measured longitudinally over two years in community-dwelling older adult men (≥65 years). Generalized estimating equations were applied to explore potential indicators by examining the association between longitudinal changes in multiple sensory and motor functions and the transition to cognitive decline. **Results**: Older age, lower uncorrected contrast sensitivity at 4 m, a lower binaural average sentence recognition score, poorer olfactory discrimination, shorter limits of stability test time, de-creased gait velocity, a reduced loading response proportion, and an increased proportion of pre-swing were related to higher odds of longitudinal cognitive decline. **Conclusions**: These results showed that specific sensory and motor functions were associated with longitudinal cognitive decline and suggested that their integration may provide information that could contribute to risk stratification for cognitive decline in older adults. Further studies with larger sample sizes and multiple cohorts are needed.

## 1. Introduction

As the proportion of the elderly population continues to increase, the prevalence of neurodegenerative disorders also rises. Among these, dementia lacks a disease-modifying cure post-onset. Therefore, early detection and preventive interventions are considered the optimal response strategies [1]. In particular, Early identification of Mild Cognitive Impairment (MCI), an intermediate stage between normal aging and dementia, plays a crucial role in delaying the future cognitive deterioration [2].

MCI is characterized by noticeable cognitive decline while the capacity to perform daily activities remains intact, indicating that the individual has not yet progressed to the stage of dementia. Cognitive decline is typically evaluated using cognitive assessment tools (e.g., MMSE, MoCA), and MCI or dementia is diagnosed by identification of probable dementia etiology through blood tests, cerebrospinal fluid analysis, MRI, CT scans, and other examinations. These conventional methods have low accessibility, are invasive, and costly. In addition, these tests are usually used only after symptoms appear, many individuals have already developed MCI or early dementia by the time they are tested. Consequently, there is an increasing demand for novel approaches that enable timely, non-invasive, and cost-effective detection of the risk of cognitive decline.

Recently, there has been increasing research on the relationship between cognitive decline and physical function. As dementia progresses, cerebral cortical atrophy occurs alongside the accumulation of brain waste products such as beta-amyloid [3,4]. This has been shown to affect not only cognitive function but also sensory and motor functions [5]. Indeed, numerous studies have observed a decline in sensory functions such as vision, hearing, and olfaction, as well as in postural balance and gait abilities, in patients with dementia and MCI. Furthermore, changes in physical function have been reported to precede cognitive decline [6,7]. The results of these studies suggest that changes in sensory and motor functions over time could potentially serve as candidate indicators of gradual cognitive decline.

However, most existing studies have been limited to analyzing sensory, balance, and gait functions individually in relation to cognitive function from a cross-sectional perspective. For instance, Rehan et al. reported a significant cross-sectional correlation between visual function disability and the risk of cognitive impairment and dementia onset [8], while Kjelvik et al. identified a clear association between impaired olfactory discrimination at a single time point and hippocampal atrophy in patients with MCI and early dementia [9]. Additionally, in cross-sectional studies of motor function, significant differences in gait parameters have been observed between Alzheimer’s patients and individuals with normal aging. Unlike previous studies, Davoudi et al. considered the association between sensory and motor functions integratively, but this research had the clear limitation of being cross-sectional in design [10].

While longitudinal studies are actively being conducted to overcome these limitations, most tend to focus predominantly on either sensory or motor functions, rather than integrating both. For instance, Wu et al. reported a longitudinal association between single and combined impairments in visual, auditory, and olfactory functions and cognitive decline [11], while Byeon et al. longitudinally confirmed that dual sensory impairment in vision and hearing is a risk factor for cognitive decline [12]. By contrast, Michelle et al., through a longitudinal analysis of gait speed and cognitive function, revealed that slow gait is a potential indicator for cognitive decline, but their analysis was limited to gait speed and did not incorporate detailed gait strategies within the gait cycle [13]. The aforementioned studies are limited because they analyzed changes in cognitive function by confining their focus to either sensory or motor function. The cognitive function of the elderly is not the independent action of either side but is closely linked to the systemic sensorimotor integration process, which includes both sensory and motor functions [14]. Within the central nervous system, sensory, somatosensory, motor, and cognitive functions are primarily represented in anatomically distinct regions, but the information processed in each region is integrated through interconnected neural networks [15,16]. Similarly, Paraskevoudi et al. reported that the brain regions involved in the integration of sensory information, higher-order cognitive functions, and motor control overlap with another [17]. These neuroanatomical characteristics support the need to consider sensory, balance, and gait functions integratively in relation to cognitive decline. Nevertheless, research tracking cognitive decline trajectories by considering both multi-sensory and motor functions remains insufficient. An integrative consideration of these functions may provide a potential means of reflecting cognitive decline trajectories. Therefore, an integrative approach may help address the limitations of existing single-domain studies and could provide useful insight into the risk of cognitive decline. In this study, cognitive decline trajectory was operationally defined as a transition from normal cognition (Korean version of the Montreal Cognitive Assessment [K-MoCA] score 26–30) to high risk for cognitive impairment (K-MoCA score of 21–25) over a 2-year follow-up.

The purpose of this study was to investigate whether integrating longitudinal changes in multiple sensory and gait functions could provide information on cognitive decline in older adults.

## 2. Materials and Methods

### 2.1. Study Design and Participants

This study used data from the longitudinal research for “the Development of Digital Diagnostics & Therapeutics for Dementia based on Machine Learning using Functions of Sensation and Gait”. The project aims to develop digital diagnostic technologies and digital therapeutics for dementia by analyzing changes in special sensory functions, postural balance, and level-ground gait function in relation to cognitive changes. The development of digital diagnostics & therapeutics for dementia project annually assesses visual, auditory, olfactory, postural balance, and level-ground walking functions in community-dwelling adults aged 65 and older.

The inclusion criteria for the development of digital diagnostics & therapeutics for dementia project were as follows: males aged 65 years or older; individuals capable of walking independently without assistance from a third party or assistive devices; those without neuromuscular diseases or residual disabilities; and those not diagnosed with dementia.

The exclusion criteria were as follows: individuals unable to walk independently, those who developed neuromuscular diseases or residual disabilities after participation, and those who developed dementia after participation.

All participants were informed about the purpose of the study, study procedures, and data collection methods, and voluntarily agreed to participate. For all participants, medical history and lifestyle patterns were collected, including neurological and musculoskeletal diseases or surgical experience, cardiovascular diseases, diabetes, hypertension, hyperlipidemia, other diseases and family history, falls, smoking and drinking status, regular exercise, and regular health check-ups. Subsequently, cognitive, sensory, postural balance, and gait function measurements were performed.

**Figure 1:**
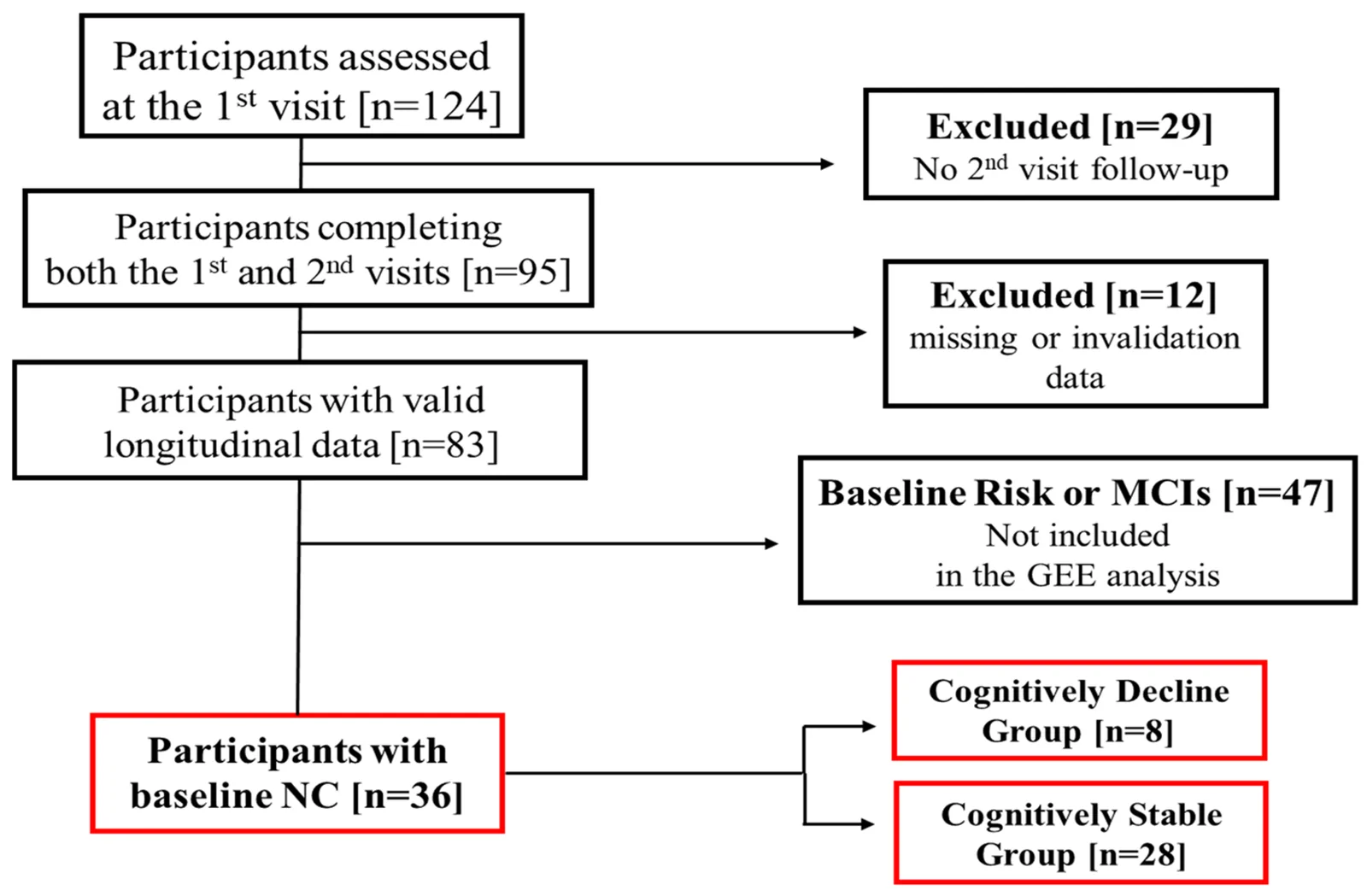
Flowchart of participant selection for the longitudinal analysis of multiple sensory, balance and gait changes associated with subsequent cognitive decline.The red box indicates the participants included in the present study.

In the first year of the project, a total of 124 individuals participated in the experiment, of whom 95 took part in the second visit. To analyze the longitudinal changes in cognition and function, this study initially selected 95 participants. Following the exclusion of 12 participants due to missing or invalid data, a final sample of 83 participants with valid longitudinal data was obtained. These 83 participants were categorized based on changes in their cognitive status between the two time points into the following groups: a cognitively stable group (*n* = 28), a cognitively declined group (*n* = 8), a group that maintained a high risk for cognitive impairment (*n* = 30), a group whose cognitive status changed from normal cognition to high risk for cognitive impairment (*n* = 14), a group that maintained suspected mild cognitive impairment or dementia (*n* = 1), and a group whose cognitive status changed from high risk for cognitive impairment to suspected mild cognitive impairment or dementia (*n* = 2). Based on the purpose of this study, the analysis focused on participants who were cognitively normal at baseline, resulting in a final analysis sample of 36 participants, comprising the cognitively stable group (*n* = 28) and the cognitively decline group (*n* = 8). The participant selection and classification processes are shown in Figure 1.

This study was conducted in accordance with the Declaration of Helsinki and received ethical approval from the Jeonbuk National University Institutional Review Board (JBNU IRB No. 2023-11-015-001).

### 2.2. Cognitive Assessment

Cognitive function was evaluated using the K-MoCA. This test comprises sections evaluating visuospatial-executive function (5 points), naming (3 points), attention (6 points), language (3 points), abstraction (2 points), delayed recall (5 points), and orientation (6 points). All segments are summed for evaluation, and for those with 12 years or less of education, 1 point is added to correct for score discrepancies due to education level.

Based on the K-MoCA score, participants were classified as having Normal Cognition (NC) if their score was 26–30 points, High Risk for Cognitive Impairment (Risk) if 21–25 points, and suspected Mild Cognitive Impairment or Dementia (MCIs) if 0–20 points [18]. In the longitudinal analysis, 36 individuals from the NC group at their first visit were categorized into a cognitively stable group (CS, those maintaining normal cognition) and a cognitively decline group (CD, those whose cognitive function declined from normal cognition to the high risk group) based on their cognitive changes over two years.

### 2.3. Gait Measurement and Analysis

**Figure 2:**
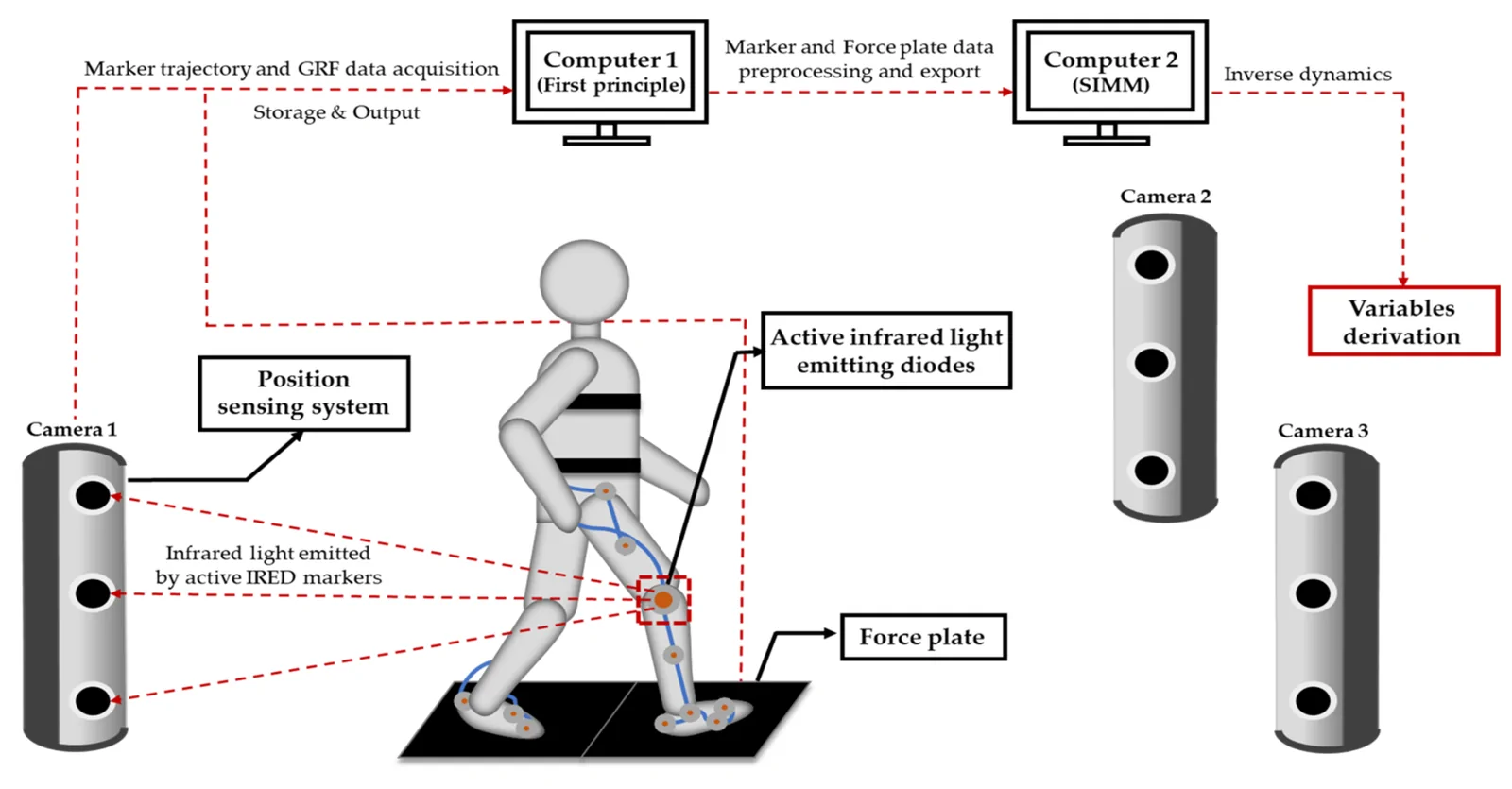
Level-ground walking measurement.

Gait function was measured by attaching 17 active infrared light emitting diodes (Smart Marker, NDI Inc., Waterloo, ON, Canada) to the participant’s body according to the Motion Module Marker Guide (MusculoGraphics, Inc., Santa Rosa, CA, USA). Marker signals during level-ground walking were collected using three position sensing systems (Optotrak Certus, NDI Inc., Waterloo, ON, Canada), and ground reaction forces were acquired using two force plates (Bertec Ltd., Columbus, OH, USA). The collected marker and force plate data were preprocessed and exported using NDI First Principles, version 1.2.4 (Northern Digital Inc., Waterloo, ON, Canada) software. Subsequently, inverse dynamics analysis was performed and gait parameters were calculated using SIMM, version 7.0 (Motion Analysis Corp., Santa Rosa, CA, USA) software. Based on a spatiotemporal function, Stride Length, Step Length, Cadence, Gait Velocity, Stride Duration, Stance Phase Duration, and Swing Phase Duration were derived. Based on the gait cycle, the proportions of Loading Response, Mid Stance, Terminal Stance, Pre-Swing, Single-Limb Support, and Double-Limb Support were calculated. The level-ground walking measurement is shown in Figure 2.

### 2.4. Balance Test

Postural balance function was evaluated using the Biodex Balance System SD (Biodex Medical Systems, Shirley, NY, USA). The evaluation included three tests: the Postural Stability Test, the Fall Risk Test, and the Limits of Stability Test (LoS).

The Postural Stability Test measures how steadily an individual maintains their body’s center. This test quantifies the degree of deviation from the center, meaning lower scores indicate superior balance ability. The calculated variables included PSI (Postural Stability Index), APSI (Anterior-Posterior Stability Index), and MLSI (Mediolateral Stability Index). The Fall Risk Test evaluates the potential risk of falling; a higher measured index indicates a higher risk of falling. The Limits of Stability Test assesses dynamic balance by actively moving and controlling the center of gravity within the base of support. From this test, the LoS Overall Direction Control Score (LoS score) and LoS Time were calculated. A higher LoS score indicates more accurate, controlled movement toward the target, in contrast to the Postural Stability Test’s index, for which higher values indicate greater deviation.

### 2.5. Sensory Measurement

#### 2.5.1. Auditory Function Test

Auditory function was measured using the Korean Speech Audiometry Test and an audiometer (GSI-61, Grason-Stadler, Eden Prairie, MN, USA). Pure Tone Audiometry (PTA), Sentence Recognition Threshold (SRT), Word Recognition Score (WRS), and Sentence Recognition Score (SRS) were tested in each ear.

Pure tone audiometry (PTA) measured pure tone thresholds at each frequency range, calculating PTA125 (125 Hz, 250 Hz, 500 Hz), PTA512 (1000 Hz, 1500 Hz, 2000 Hz), and PTA468 (4000 Hz, 6000 Hz, 8000 Hz) bands. For each PTA band, the analysis variables were set as the average pure tone threshold of both ears, the difference in pure tone thresholds between the both ears, and the better pure tone threshold of the both ears.

For SRT, the analysis variables were the mean speech recognition threshold of both ears, the difference in speech recognition threshold between the two ears, and the better speech recognition threshold.

For WRS, the analysis variables were the difference in word recognition scores, the mean word recognition score, the better word recognition score, and the number of incorrect word recognitions.

For SRS, the analysis variables were the mean sentence recognition score for both ears, the difference in sentence recognition scores, the better sentence recognition score, and the number of incorrect sentence recognitions.

#### 2.5.2. Olfactory Function Test

Olfactory function was evaluated using the Snap & Sniff Olfactory Test Kit (Sensonics International, Haddon Heights, NJ, USA), which consisted of three subtests: Threshold, Discrimination, and Identification.

The Olfactory Threshold Test assesses the minimum concentration of an odor that can be detected. Participants choose a numbered scent from two options, with higher scores indicating the ability to detect lower concentrations. The Olfactory Discrimination Test assesses the ability to distinguish between different odors. This test involves identifying the one scent that is different among three options. Finally, the Olfactory Identification Test evaluates the ability to recognize presented odors by having participants smell a scent and identity it from provided selections.

For the first test, the Threshold was set as the analytical variable, while the number of correct answers was used for both the Discrimination Test and the Identification Test.

#### 2.5.3. Visual Function Test

Visual function was measured using two tests: minimum readable threshold and contrast sensitivity. These were performed using a standard international vision chart and a Low Contrast Flip Chart (Lea Numbers 10M Flip Chart, Lea Test international. LLC, Etters, PA, USA), respectively.

Visual acuity measured the unaided vision of both eyes at a distance of 4 m from the vision chart, following the test guidelines. Contrast sensitivity was also measured for both eyes without correction at distances of 4 m, 3 m, 2 m, and 1.6 m, according to guidelines. For the minimum readable threshold, the average visual acuity of both eyes, the difference in visual acuity, and the better visual acuity were analyzed. For contrast sensitivity, the number of correct answers at each distance, the total number of correct answers, and the average number of correct answers were analyzed.

### 2.6. Statistical Analysis

GEE were used to explore potential indicators related to progressive cognitive decline. Subsequently, differences in the longitudinal changes of the selected indicators were analyzed. There was an imbalance in sample size between the two groups, with 28 participants in the cognitively stable group and 8 in the cognitively decline group. Therefore, non-parametric statistical tests were conducted. The Mann-Whitney U test was used to identify differences between the two groups each year, and the Wilcoxon-signed-rank test was performed to identify significant changes within groups over two years. The significance level was set at *p* < 0.05, and all statistical analyses were conducted using SPSS 27.0 software (IBM Corp., Armonk, NY, USA).

## 3. Results

### 3.1. Demographic Characteristics

The baseline demographic characteristics of the study participants were presented in Table 1.

A significant difference between the two groups was observed only in age (*p* = 0.044). The mean age of the CS group was 2.24 years younger than the CD group. No significant differences were found between the two groups in terms of educational attainment, height, weight, or BMI. This suggests an association between age and cognitive function indicating that older age can be a risk factor for cognitive decline.

### 3.2. Exploration of Candidate Indicators Associated with Cognitive Decline

GEE were used to derive longitudinal candidate indicators associated with cognitive decline, with cognitive status coded as 0 for the CS group and 1 for the CD group. Thus, the CS group served as the reference category. A binomial distribution with a logit link function was specified, and an independent working correlation structure was used for the repeated measurements. Age was included as a covariate in the model to adjust for its potential influence.

The candidate indicators related to longitudinal cognitive decline are presented in the Table 2. The investigated indicators related to cognitive decline were Limits of Stability (LoS) test time, Contrast Sensitivity at 4 m with Uncorrected Vision (Un_4mCS), Binaural Average Sentence Recognition Score (SRS_Avg), Olfactory Discrimination (Olf_ Disc), Gait Velocity (Velocity), Loading Response Proportion (LR), Pre-Swing Proportion (PS) and Age. With age included as a covariate in the analysis, Longer LoS test time, increased Un_4mCS, higher SRS_Avg, improved Olfactory Discrimination, and increased Velocity and LR were associated with lower odds of cognitive decline. Conversely, increased PS and Age were associated with higher odds of cognitive decline.

### 3.3. Candidate Factors in Sensory Function Variables

The longitudinal changes in sensory functions derived as candidate factors related to cognitive decline are shown in Table 3.

At the first visit, a significant between-group difference was observed in visual function. The CS group exhibited significantly higher Un_4mCS compared to the CD group. This suggests that the group with future cognitive decline already showed lower Un_4mCS from the outset. Similar findings were observed in auditory and olfactory function. Although not reaching statistical significance, SRS_Avg and Olf_Disc in the CD group were also observed to be lower than those in the CS group at the first visit.

### 3.4. Candidate Factors in Balance and Gait Function Variables

The longitudinal changes in the postural balance and gait features derived as candidate factors are shown in Table 4.

Regarding balance function, the CS group showed a significant increase in LoS score (*p* = 0.029) with little change in LoS time. This indicates an improvement in dynamic balance accuracy in the group that maintained normal cognitive function. Regarding gait function, the LR, the initial stance phase, significantly increased in the CD group (*p* = 0.012), and the PS, a late stance phase, also showed an increasing trend. These two phases are key components of the Double Limb Support Proportion (DLSP) during gait. The notable increase in LR led to a significant increase in DLSP (*p* = 0.025). This suggests that when cognitive function deteriorates, there is a compensatory tendency arises to increase DLSP, particularly LR, which is the weight acceptance phase, in order to maintain stability while walking.

## 4. Discussion

In the present study, we explored the potential of the longitudinal changes in multiple sensory and motor functions, considered collectively, to provide information on cognitive decline in older adults.

Age was included as a covariate, with increasing age being associated with higher odds of cognitive decline. This finding aligns with existing studies indicating that age is one of the most significant risk factors for cognitive impairment [19]. Caroline N. Harada et al. reported that neurodegenerative disease-related changes, such as cerebral atrophy, white matter degradation, and the accumulation of neuropathological proteins, progressively occur even during the course of normal cognitive aging [20]. This suggests that normal cognitive aging and neurodegenerative diseases are not entirely independent processes, but rather that the underlying biological mechanisms driving these changes may largely overlap or exist on a continuum [21]. Consequently, increased age causes an accumulation of biological changes, which can ultimately heighten vulnerability to cognitive decline.

Changes in cognitive function are also closely related to declines in multiple sensory functions such as vision, hearing, and olfaction. Among visual functions, as Un_4mCS increased, the odds of cognitive decline decreased. This supports previous research indicating that a decrease in contrast sensitivity function increases the risk of developing MCI or dementia [22], and can be explained by the significant association of contrast sensitivity with amyloid and tau accumulation, as well as temporal lobe atrophy and neurodegenerative changes [23].

In olfactory function, as Olf_Disc improved, the odds of cognitive decline decreased. Olfactory discrimination function is closely linked to cognitive function, which is reported as a sensitive indicator for detecting cognitive decline because it reflects complex cognitive processing [24]. Given that amyloid-beta accumulation appears first in the olfactory bulb, a decline in olfactory function may indicate an early sign of Alzheimer’s pathology [25].

Regarding auditory function, as the SRS_Avg increased, the odds of cognitive decline decreased. Numerous previous studies have also suggested that hearing impairment is a major factor promoting cognitive decline [26,27,28], as explained by the information-degradation hypothesis [29]. Specifically, excessive compensatory cognitive resources are allocated to processing degraded auditory signals, leading to the depletion of overall higher cognitive functions.

Not only sensory functions, but also subtle changes in balance and gait play a critical role as candidate factors related to cognitive decline. For balance function, as LoS time lengthened, the odds of cognitive decline decreased. According to Table 4, the CD group exhibited a decrease in LoS time and less improvement in accuracy compared to the CS group. Conversely, the CS group showed almost no change in LoS time, but their LoS score increased significantly, indicating improved accuracy in performing balance tasks. Balance control is a complex function that combines sensory integration, motor planning, and cognitive processes such as attention and executive function [30,31]. Cognitive changes influence postural control [32], and performance patterns on balance tasks may vary depending on changes in cognitive function [33].

For gait function, as Velocity increased, the odds of cognitive decline decreased. Meanwhile, the detailed gait cycle parameters of LR and PS revealed opposing trends. Specifically, an increased LR was associated with decreased odds of cognitive decline, whereas an increased PS was associated with increased odds of cognitive decline.

Gait and cognitive function are interconnected processes, linked by shared neural circuits that integrate sensory information and facilitate the planning and modulation of movement. These neural networks involve temporoparietal and frontal motor regions, which interact with the basal ganglia, cerebellum, and brainstem to regulate posture and gait [34].

Gait can be understood in terms of pace and rhythm. Pace includes parameters such as velocity, cadence, stride length, and step length, and reflects the overall efficiency of gait. Pace has been reported to be associated with gray matter volume (GMV) in the bilateral supplementary motor areas (SMA), the right superior temporal sulcus, and bilateral cerebellar regions, which encompass neural circuits involved in processing multisensory information as well as in cognitive and automatic gait control [35]. Rhythm is related to the temporal characteristics of the gait cycle and has been reported to be associated with the GMV in the bilateral SMA, cingulate, prefrontal, and paracingulate cortices [33]. SMA is known to be involved in the regulation of the sequence and timing of movements [36], and the prefrontal cortex is involved in cognitive processes such as attention and executive function [37]. Therefore, rhythm can reflect the temporal organization of each stage. This may also be reflected in the phase-specific temporal characteristics of each stage within the gait cycle. In this context, LR reflects the features of the early stance phase, characterized by weight acceptance and postural stabilization, whereas PS reflects those of the terminal stance phase, characterized by propulsion and preparation for the transition to the swing phase. Given that gait and executive function are controlled by similar neural circuits, the allocation of resources and performance strategies required at each stage of the gait cycle may differ depending on cognitive function status [38]. Furthermore, recent clinical investigations evaluating complex motor and balance interventions have demonstrated that targeted improvements in motor control yield measurable parallels in functional and cognitive outcomes [39,40], highlighting the profound therapeutic and diagnostic interconnectedness of the sensorimotor and central nervous systems.

In summary, this study suggests that gradual changes in cognitive function in older adults are not limited to a single sensory or motor domain but may be accompanied by changes across multiple sensorimotor domains. Thus, considering these changes from an integrated perspective may provide useful insights into the relationship between cognitive and sensorimotor function. These findings should be interpreted in light of the modest sample size, particularly the limited number of participants in the cognitive decline group. In clinical practice, these findings may have potential as a supplementary approach to risk-stratification, helping to inform a decision about whether older adults may benefit from further clinical evaluation, such as MRI or cerebrospinal fluid analysis.

## 5. Limitations

Several limitations exist in this study. First, the study participants were limited to males. Previous studies suggest that there may be sex differences in the mechanisms and manifestations of cognitive decline [41,42], highlighting the necessity of considering sex-specific characteristics in the early detection of cognitive impairments. Further studies should include female participants and adopt designs that adequately account for sex-related characteristics.

Second, this study lacks external validation. Because the data were derived from a single cohort within a specific geographic region, there are limitations regarding the generalizability of the findings to other populations or geographic areas. Future studies should incorporate multicenter, geographically diverse cohorts to perform external validation, thereby confirming the reproducibility and generalizability of these findings.

Third, the two-year follow-up period was relatively short and may be insufficient to fully capture the long-term cognitive trajectory. Nevertheless, even within this limited timeframe, we examined associations between changes in sensory, balance, and gait functions and cognitive decline trajectory. In the future studies, Future follow-up period should be extended to more clearly capture the progression of cognitive decline and its temporal relationship with changes in physical function.

Fourth, the study was limited by a small sample size and an imbalance in sample sizes across cognitive function groups. These limitations may have reduced statistical power and increased the risk of Type II errors, as well as the potential for unstable parameter estimates or overfitting when simultaneously accounting for multiple predictor variables. Therefore, future studies should include a larger, more balanced sample to enhance statistical power and improve the reliability of the estimated associations.

Fifth, cognitive function was assessed using only the K-MoCA. Future studies should incorporate a broader battery of neuropsychological assessments to enable a more precise assessment of cognitive function and its changes over time.

## 6. Conclusions

This study examined longitudinal changes in multiple sensory and motor functions and explored their associations with subsequent cognitive decline in older adults.

Our findings show that a decrease in Un_4mCS in visual function, a decrease in SRS_Avg in auditory function, a worsening of Olf_Disc in olfactory function, a shorter LoS time in dynamic balance, and decreases in Velocity and LR in gait was related to higher odds of cognitive decline. Conversely, increased PS in gait function and older Age as a covariate was related to higher odds of cognitive decline. In other words, contrast sensitivity, sentence recognition, olfactory discrimination, the LoS test, and spatiotemporal and gait-phase analyses may be considered informative measures in visual, auditory, olfactory, postural balance, and gait function, respectively.

These results imply that specific longitudinal changes in sensory and motor functions are associated with cognitive decline over the follow-up period and may provide additional insight beyond previous studies that have primarily examined sensory or motor functions separately, often using cross-sectional designs. These findings suggest that longitudinal changes in multiple sensory and motor functions, when considered collectively, may have potential as indicators of subsequent cognitive decline in older adults.

The explored candidate factors may offer a low-cost, and non-invasive approach to risk stratification in older adults at risk of cognitive decline. They may also provide useful information to support further clinical evaluation and follow-up. Further studies with larger sample sizes, multiple independent cohorts, and consideration of sex differences are needed to validate these findings and assess their generalizability.

## Figures and Tables

**Table 1:** Participant’s Characteristics.

Group	Age (yrs)	Education (yrs)	Height (cm)	Weight (kg)	BMI (kg/m^2^)
CS	73.64 ± 3.74 *	14.79 ± 2.81	166.19 ± 5.59	67.03 ± 7.17	24.27 ± 2.30
CD	75.88 ± 4.02 *	15.00 ± 2.14	166.60 ± 5.16	67.58 ± 6.55	24.33 ± 1.93
Total	74.14 ± 3.86	14.83 ± 2.65	166.28 ± 5.43	67.15 ± 6.95	24.29 ± 2.20

Mean ± Standard deviation; CS: Cognitively Stable, normal cognition at both 1st and 2nd visits, CD: Cognitively Decline, normal cognition at 1st visit and high-risk of cognitive impairment at 2nd visit. * Indicates significance difference between two groups via Mann-Whitney U Test, *p*-value < 0.05.

**Table 2:** Candidate Indicators for transition to Cognitive Decline.

Parameters	B	S.E.	Wald χ^2^	OR	95% CI	*p* Value
Age *	1.620	0.7984	4.118	5.054	1.057–24.169	0.042
Un_4mCS *	−1.693	0.5508	9.443	0.184	0.063–0.542	0.002
SRS_Avg *	−1.357	0.4818	7.930	0.258	0.100–0.662	0.005
Olf_Disc *	−1.203	0.3743	10.330	0.300	0.144–0.625	0.001
LoS time *	−1.349	0.5793	5.423	0.259	0.083–0.808	0.020
Velocity *	−1.768	0.6832	6.699	0.171	0.045–0.651	0.010
LR *	−3.347	0.8632	15.036	0.035	0.006–0.191	<0.001
PS *	2.479	0.8804	7.925	11.924	2.123–66.963	0.005

Reference group: CS group; Un_4mCS: Contrast Sensitivity at 4 m with Uncorrected Vision; SRS_Avg: Binaural Average Sentence Recognition Score; Olf_Disc: Olfactory Discrimination; LoS time: Limits of Stability test time; LR: Loading Response Proportion; PS: Pre-Swing Proportion; Age was included as a covariate in the GEE. * indicates statistical significance via the generalized estimating equations (GEE) analysis, *p* < 0.05.

**Table 3:** Two-Year Longitudinal Changes of Multiple Sensory Functions by Cognitive Group.

Group	Visit	Un_4mCS	SRS_Avg	Olf_Disc
CS	1st	10.4 ± 7.6 *	93.8 ± 6.5	13.8 ± 3.2
	2nd	10.1 ± 6.9	93.0 ± 8.2	13.8 ± 3.5
CD	1st	4.5 ± 5.4 *	91.9 ± 5.9	10.6 ± 3.7
	2nd	5.3 ± 4.9	87.5 ± 11.0	12.1 ± 3.2

Mean ± Standard deviation; CS: Cognitively Stable, normal cognition at both 1st and 2nd visits; CD: Cognitively Decline, normal cognition at 1st visit and high-risk of cognitive impairment at 2nd visit. Un_4mCS: Contrast Sensitivity at 4m with Uncorrected Vision; SRS_Avg: Binaural Average Sentence Recognition Score; Olf_Disc: Olfactory Discrimination. * indicates significance difference between the two groups via Mann-Whitney U Test, *p* < 0.05.

**Table 4:** Two-Year Longitudinal Changes of Balance and Gait Functions by Cognitive Group.

Group	Visit	LoS Score	LoS Time	Velocity	LR	PS	DLSP
CS	1st	33.0 ± 9.2 ^†^	51.7 ± 12.5	125.1 ± 15.2	10.5 ± 1.4	10.9 ± 1.2	21.4 ± 2.4
	2nd	36.6 ± 9.7 ^†^	51.6 ± 17.6	124.8 ± 16.8	10.7 ± 1.3	11.1 ± 1.3	21.7 ± 2.6
CD	1st	31.6 ± 10.6	52.6 ± 19.0	121.6 ± 17.0	10.1 ± 0.9 ^†^	10.9 ± 1.2	21.0 ± 1.8 ^†^
	2nd	34.4 ± 9.2	50.3 ± 14.4	120.1 ± 17.2	10.7 ± 1.0 ^†^	11.1 ± 0.8	21.8 ± 1.5 ^†^

Mean ± Standard deviation; CS: Cognitively Stable, normal cognition at both 1st and 2nd visits; CD: Cognitively Decline, normal cognition at 1st visit and high-risk of cognitive impairment at 2nd visit. LoS score: Limits of Stability test overall direction control score; LoS time: Limits of Stability test time; LR: Loading Response Proportion; PS: Pre-Swing Proportion; DLSP: Double Limb Support Proportion; ^†^ indicates significance changes in first and second visits via Wilcoxon Sign-Rank Test, *p* < 0.05.

## Data Availability

The datasets presented in this article are not readily available because the data are part of an ongoing research project. Requests to access the datasets should be directed to the corresponding author.

## Abbreviations

The following abbreviations are used in this manuscript:

MCI	Mild Cognitive Impairment
MMSE	Mini-Mental State Examination
MoCA	Montreal Cognitive Assessment
CS	Cognitively Stable
CD	Cognitively Decline
LoS	Limits of Stability test
LoS score	Limits of Stability overall direction control score
PTA	Pure Tone Audiometry
SRT	Sentence Recognition Threshold
WRS	Word Recognition Score
SRS	Sentence Recognition Score
Un_4mCS	Contrast Sensitivity at 4 m with Uncorrected Vision
SRS_Avg	Binaural Average Sentence Recognition
Olf_Disc	Olfactory Discrimination
LR	Loading Response Proportion
PS	Pre-Swing Proportion
DLSP	Double Limb Support Proportion
